# Incidental Finding of Well-Differentiated Duodenal Neuroendocrine Tumor on Diagnostic Upper Endoscopy

**DOI:** 10.7759/cureus.53568

**Published:** 2024-02-04

**Authors:** Kelsey Lamb, Shiv B Gakhar, Suresh Jayatilaka

**Affiliations:** 1 Internal Medicine, Edward Via College of Osteopathic Medicine, Blacksburg, USA; 2 Gastroenterology and Hepatology, Edward Via College of Osteopathic Medicine, Blacksburg, USA; 3 Gastroenterology and Hepatology, Sentara Halifax Regional Hospital, South Boston, USA

**Keywords:** gastrointestinal carcinoid tumor, hyperplastic polyp, duodenal bulb, duodenal carcinoid, gastrointestinal neuroendocrine tumor

## Abstract

Neuroendocrine tumors (NETs) are rare and slow-growing. They are often found incidentally, and patients typically present with vague symptoms. This is a case report detailing an 83-year-old female who presents with signs and symptoms consistent with esophageal stricture and was incidentally found to have a duodenal NET. Treatment typically involves surgical removal and carries a good prognosis. With complete surgical resection of localized tumors, the chance of progression or recurrence is low.

## Introduction

Neuroendocrine tumors (NETs), formerly known as carcinoid tumors, are predominantly located in the gastrointestinal tract. Among this specific group, duodenal NETs are infrequently encountered. With that being said, duodenal NETs make up approximately 2% of all gastrointestinal neoplasms [1]. These tumors may present asymptomatically or symptomatically depending on location. When small in size, NETs are typically found incidentally and patients remain asymptomatic. Larger tumors may present with nonspecific signs such as nausea, vomiting, and/or abdominal pain. Carcinoid syndrome may also be present upon evaluation. NETs arise from neuroendocrine cells; thus, they can be found in the appendix, lungs, pancreas, liver, and digestive tract. A population-based study done in 2017 designed to obtain a more robust epidemiologic survey of NETs originating in the small bowel found 4,130 patients of 54,764,180 individuals over an 18-year period to have small bowel NETs. This calculates out to an overall prevalence of 7.54/100,000 persons [2]. The diagnosis is based on histology and confirmed via immunohistochemical staining. NETs characteristically stain positive for one or more neuroendocrine tumor markers, such as synaptophysin or chromogranin A [3]. We report a case of a patient with a neuroendocrine tumor found in the duodenal bulb on diagnostic upper endoscopy who presented with complaints of recurrent dysphagia and persistent gastroesophageal reflux disease (GERD). The diagnosis was confirmed via immunohistochemical staining. The patient was referred to oncology after endoscopic polypectomy. 

## Case presentation

An 83-year-old Caucasian female with a past medical history of irritable bowel syndrome with constipation (IBS-C), GERD, type 2 diabetes mellitus, hypertension, hyperlipidemia, hypothyroidism, and obstructive sleep apnea presented to the gastroenterology office with complaints of solid food dysphagia for approximately three months. She denied nausea, vomiting, abdominal pain, unintentional weight loss, hematochezia, or melena. Her IBS has been well controlled on linaclotide. At that time, the patient also noted severe GERD on dexlansoprazole, famotidine, and sodium alginate-bicarbonate. She had no relief with omeprazole in the past. She had an esophagram done previously that showed moderate presbyesophagus and poor relaxation at the lower esophageal sphincter. The patient reported having one prior esophagogastroduodenoscopy (EGD) done remotely that was normal. A diagnostic EGD was scheduled and performed a few weeks after her initial visit and showed a stricture at the gastroesophageal junction, salmon mucosa, a medium hiatal hernia, gastritis, a single gastric polyp, and a single duodenal polyp. The stricture was dilated, and biopsies were taken. Pathology report returned as hyperplastic gastric polyp and duodenal adenoma. However, biopsy samples taken from the duodenal polyp were insufficient for further testing.

**Figure 1:**
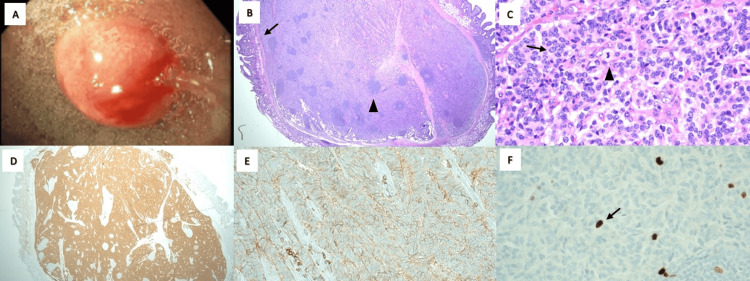
Histopathology of duodenal neuroendocrine tumor (A) Six-millimeter duodenal polyp found on upper endoscopy with biopsies returning positive for well-differentiated neuroendocrine tumor. (B) Low-power cross-sectional view of a neuroendocrine tumor with surrounding clear margins and small bowel epithelium seen at the surface (black arrow), benign lymphoid aggregates (black arrowhead) seen throughout (H&E, 40x, duodenal polyp biopsy). (C) Higher-power view displaying the characteristic nuclear features with a “salt and pepper” chromatin appearance (black arrow) and a single mitotic figure (black arrowhead) (H&E, 200x, duodenal polyp biopsy). (D) Synaptophysin stain shows diffuse nuclear staining, supportive for well-differentiated neuroendocrine tumor (IHC, 200x, duodenal polyp biopsy). (E) Positive CD56 immunohistochemical stain, further supporting diagnosis (IHC, 200x, duodenal polyp biopsy). (F) Ki-67 stain (black arrow), a biological tumor marker of active cell proliferation, was < 3% indicating a low-grade (G1) or well-differentiated tumor (IHC, 200x, duodenal polyp biopsy). H&E: hematoxylin and eosin, IHC: immunohistochemistry.

Upon follow-up, the patient noted a 1.6-pound weight loss. She affirmed improvement in dysphagia after dilation and stated that her GERD has been well controlled on her current medication regimen. A repeat EGD was scheduled for complete resection of the duodenal adenoma. EGD findings included one benign-appearing, intrinsic mild stenosis at the gastroesophageal junction. The stenosis was traversed, and dilation with an 18-19-20 mm balloon dilator was performed to 20 mm. A medium-sized hiatal hernia was present. A single 10 mm sessile polyp was found at the pylorus. The polyp was removed with a piecemeal technique using a hot snare. A single sessile polyp was found in the duodenal bulb. The polyp was removed via hot snare. Resection and retrieval were complete. Specimens were sent to pathology (Figure 1).

Per pathology report, the polyp at the pylorus was found to be a hyperplastic polyp with no evidence of malignancy or dysplasia. Giemsa stain was negative for *Helicobacter pylori*. The duodenal polyp status post polypectomy revealed a 6-mm well-differentiated grade 1 (G1) neuroendocrine tumor. The tumor cells stained positive for synaptophysin, chromogranin A, CD56, and pancytokeratin (focally). Ki-67 labeling index was less than 3% (Figure 1). The tumor was completely excised and was 0.5 mm from the margin of resection. At the follow-up appointment, the patient reported a 1.4-pound weight gain and further improvement in dysphagia. She denied any current complaints at that time. The patient was referred to oncology for further evaluation/management and to discuss treatment options.

## Discussion

Neuroendocrine tumors have the greatest incidence in the gastrointestinal tract (55%) and the bronchopulmonary system (30%). Other locations include the pancreas, liver, kidneys, and prostate. Within the gastrointestinal tract, most NETs occur in the small intestine (45%), followed by the colon, appendix, and stomach [4]. Gastrointestinal neuroendocrine tumors with a primarily duodenal location are rare clinical entities that represent less than 2% of all GI NETs. These are historically small, solitary lesions with a majority ranging from 2 mm to 3 cm in size. Approximately 90% arise in the first and second part of the duodenum. In our case, a singular 6-mm nodular lesion was present in the proximal duodenum (Figure 1A).

Clinical presentation is often nonspecific with the primary complaint being abdominal discomfort secondary to the indolent nature of NETs. If the tumor is localized near the duodenal bulb, other symptomatic complaints can include nausea, vomiting, and diarrhea. Painless obstructive jaundice is more common among periampullary tumors. Carcinoid syndrome is the most common systemic manifestation of carcinoid tumors; however, this presentation is not a common finding in rectal, duodenal, and appendiceal neuroendocrine tumors [5]. Carcinoid syndrome presents with symptoms such as nausea, vomiting, flushing, abdominal pain, diarrhea, and wheezing. These symptoms occur due to the increase in vasoactive amines including serotonin, kinins, and prostaglandins. In severe cases, this can lead to carcinoid crisis and serotonin-mediated fibrotic cardiac disease. As mentioned previously, carcinoids located in the duodenum carry a low risk for carcinoid syndrome and/or crisis; thus, our patient presentation was more nonspecific.

Due to differing presentations, initial diagnosis is often difficult. The indolent nature of NETs and similar appearance to benign polyps poses a limitation to early detection and diagnosis of NETs. Measuring serum chromogranin A and urinary excretion of 5-hydroxyindoleacetic acid (5-HIAA) is recommended for those with vasoactive symptomatology. Patients who present primarily with abdominal symptoms, as in our case, can first be evaluated with imaging modalities; however, the gold standard assessment is done with an EGD biopsy to confirm the diagnosis. Immunohistochemical staining is required for diagnosis [6]. Traditional neuroendocrine markers include synaptophysin and chromogranin A, both of which were positive in our patient. Carcinoids classically display strong, diffuse positivity for both markers.

Duodenal NETs carry a good prognosis. The preferred treatment modality is endoscopic removal with full margin excision. Characteristically, tumors less than 2 cm have strong curability with endoscopic resection. NETs larger than 2 cm may require a full-thickness resection along with regional lymphadenectomy [7]. Post-removal recommendations for the tumor include frequent endoscopic follow-ups to ensure no recurrence of the lesion. Metastatic NETs are treated with a combination of excision and somatostatin analog medication for symptomatic control. Furthermore, gastrointestinal NETs have a low chance of progression and recurrence with early surgical intervention.

## Conclusions

We present a case of an incidental finding of a well-differentiated duodenal neuroendocrine tumor (NET) during diagnostic upper endoscopy, emphasizing the elusive nature of its symptoms. Our discussion delves into the nuances of diagnosing and managing duodenal NETs, highlighting the significance of early surgical intervention. This case report underscores the need to consider duodenal NETs as a potential differential for patients with nonspecific gastrointestinal symptoms. These types of tumors can often lead to nonspecific symptoms, making their initial diagnosis challenging. Our patient initially presented with vague symptoms such as dysphagia and GERD, which were likely secondary to esophageal stricture. The incidental finding of her duodenal NET mirrors the indolent nature of duodenal NETs, making early detection crucial for successful outcomes. Initial investigations, including endoscopy and imaging, may not always reveal the presence of these tumors, necessitating repeated evaluations and careful consideration of less common differential diagnoses.

## References

[1] Malladi UD, Chimata SK, Bhashyakarla RK, Lingampally SR, Venkannagari VR, Mohammed ZA, Vargiya RV (2023). Duodenal neuroendocrine tumor-tertiary care centre experience: a case report. World J Transl Med.

[2] Saleh MA, Mansoor E, Anindo M, Isenberg G (2017). The prevalence of carcinoid tumors of the small intestine in the USA: a population-based study: 2017 Lawlor resident award: 2017 presidential poster award. Am J Gastroenterol.

[3] Pinchot SN, Holen K, Sippel RS, Chen H (2008). Carcinoid tumors. Oncologist.

[4] Naalla R, Konchada K, Kannappan O, Lingadakai R (2014). Duodenal carcinoid with carcinoid syndrome. BMJ Case Rep.

[5] Wandhare T, Guerrero M, Madlinger R, Zuberi J (2020). A rare case of duodenal carcinoid tumors in a patient with atypical symptoms. J Curr Surg.

[6] Robertson RG, Geiger WJ, Davis NB (2006). Carcinoid tumors. Am Fam Physician.

[7] Dhaduk VR, Johri V, Majesty SR, Mushtaque N, Jain N, Reddy PK (2020). Laparoscopic resection of duodenal carcinoid: a feasible method: single institute case series. J Minim Access Surg.

